# Long-Term Outcomes in Primary Obstructive Megaureter Treated by Endoscopic Balloon Dilation. Experience After 100 Cases

**DOI:** 10.3389/fped.2018.00275

**Published:** 2018-10-05

**Authors:** Ruben Ortiz, Alberto Parente, Laura Perez-Egido, Laura Burgos, José Maria Angulo

**Affiliations:** Pediatric Urology, Hospital General Universitario Gregorio Marañón, Madrid, Spain

**Keywords:** primary obstructive megaureter, endoscopic balloon dilation, long term, ureteral obstruction, hydroureteronephrosis

## Abstract

**Aim:** To assess long-term effectiveness, complications, and outcomes of primary obstructive megaureter (POM) treated by endoscopic balloon dilation (EBD) in the largest series reported.

**Patients and Methods:** Hundred POM in 92 consecutive patients were treated by EBD between years 2004 and 2016. A total of 79 POM (73 patients) with more than 18 months of follow-up after treatment have been analyzed. EBD of the vesicoureteral junction was performed with semicompliant high-pressure balloon catheters (2.7FG) with minimum balloon diameter of 5 mm, followed by temporary Double-J stent placement. Follow-up protocol included periodical clinical reviews, US and MAG-3 renogram scans.

**Results:** Median age at surgery was 4 months (15 days−3.6 years), with median operating time of 20 min (10–60) and hospital stay of 1 day (1–7). Initial renal function was preserved in all patients with significant improvement in renal drainage on the MAG-3 diuretic renogram after endoscopic treatment (*p* < 0.001 *T*-test). Significant post-operative differences were observed in hydronephrosis grade and ureteral diameter that were maintained in the long-term (*p* < 0.001 *T*-test). Endoscopic approach of POM had a long-term success rate of 87.3%, with a mean follow-up of 6.4 ± 3.8 years. Secondary VUR was found in 17 cases (21.5%), being successfully treated by endoscopic subureteral injection in 13 (76.4%). Nine cases developed long-term re-stenosis (12.2%) that were successfully treated with a new EBD in 8. Endoscopic management of POM failed in 10 cases (12.7%) that required ureteral reimplantation. Five were early failures (4 intraoperative technical problems and 1 double-J stent migration with severe re-stenosis), and 5 long-term (4 persistent VUR and 1 re-stenosis recurrence).

**Conclusion:** EBD has shown to be an effective treatment of POM with few complications and good outcomes at long-term follow up. Main complication was secondary VUR that could also be treated endoscopically with a high success rate. In our opinion, EBD may be considered first-line treatment in POM.

## Introduction

Primary obstructive megaureter (POM) is a well-known entity in pediatric urology. Most patients only need conservative management since functional obstruction resolves spontaneously in most cases, during the first months of life, without renal impairment or symptoms (1). Surgical treatment is then reserved for those cases who develop progressive hydro-ureteronephrosis with urinary tract infections (UTI) and/or renal function loss. However, its management and therapeutic options remain controversial. Ureteral reimplantation with or without ureteral tapering has been considered the gold-standard procedure for these patients, but in small infants, reimplantation of a huge ureter is challenging and it entails to potential complications (2).

Therefore, less-invasive procedures such endoscopic treatments have been proposed as alternative options in the initial management of POM, becoming so popular in the last years.

Endoscopic balloon dilation (EBD) of the vesicoureteral junction (VUJ) was first described by Angulo et al. in 1998 as initial approach of complicated POM (3). Since then several publications have shown that EBD using the original technique or a variation of the same principle is feasible, safe, and a real less-invasive procedure even for very young patients (4–8). During last years the interest has been focused on the long term effectiveness of this procedure, based on the good long-term results and suggesting that EBD could be a valid option as the definitive treatment of POM (9–11). However, it is difficult to establish its validity as a definitive treatment since only short series of patients have been reported.

In 2004, we established in our institution the EBD of the VUJ and temporary stenting as first treatment option in POM with surgical criteria. After all these years performing the technique we hypothesize that is a valid option as definitive treatment in POM; then, we wanted to assess its long-term effectiveness, complications, and outcomes in the longest series reported along with an extensive period of follow-up.

## Patients and methods

One hundred of POM in 92 consecutive patients were treated by EBD between years 2004 and 2016. A total of 79 POM in 73 patients (6 patients had bilateral POM) with more than 18 months of post-operative follow-up were retrospectively analyzed. We excluded for the analysis 8 patients with shorter follow-up, and 11 patients who were referred from other institutions, and after initial treatment follow-up was carried out in their referrals. Fifty-eight were boys (80%) and 15 girls (20%), with affection of the left side in 51p (64.6%) vs. 22p right side (27.8%), and 6 bilateral (7.6%). Prenatal US diagnosis was done in 56p (76.7%).

Diagnosis and management of POM was done according to the European guidelines and consensus statement of this entity. Primary obstructive megaureter was considered in those that presented progressive hydro-ureteronephrosis with distal ureter diameter greater than 10 mm, obstructive pattern on MAG3 diuretic renogram scan and absence of vesicoureteral reflux on cystography.

US was used to measure the diameter of pelvis, calyces, distal ureter, and the characteristics of renal parenchyma. Hydro-ureteronephrosis grade was defined according to the guidelines of the Society of Fetal Urology. It was done at birth (in cases of prenatal diagnosis), at 1 month of life and then every 3 months under conservative surveillance with low-dose antibiotic prophylaxis. Mercaptoacetyltriglycine (MAG-3) renal scans with furosemide washout revealed obstructive pattern with progressive cumulative radiotracer in ureteral area in all cases. Good urinary drainage out of the regions of interest at 30 min after injection of the MAG-3 without diuretic test was considered as no obstruction. If poor drainage was detected, a diuretic test was performed (intravenous furosemide 1 mg/kg) and total urinary drainage was calculated during the 20 min after the injection. Washout halftime T1/2 > 20 min after furosemide injection was defined as an obstruction. Changes in patient position and postmicturition imaging are included for the analysis of the renography results. In our institution, when the half-life of the isotope is estimated > 50 min, it is determined as T1/2 > 50 min. All patients presented obstructive diuretic renogram T1/2 > 50 min with mean differential function of the affected kidney 44.4% ± 6.3.

Nevertheless, not all of these patients needed surgical repair (in our series only 13% of cases prenatally diagnosed). The indication for surgical intervention was established in those with one or more of the following conditions (Table 1):

- Breaking-through febrile UTI in 30 cases (38%) despite antibiotic prophylaxis, with clinical scenario of pyonephrosis and sepsis in 6 patients at time of treatment.
- Progressive worsening of hydro-ureteronephrosis with renal parenchyma thinning in 29 cases (36.7%).
- Impairment of renal function (differential renal function < 40% at diagnosis or decreasing more than 10% during expectative surveillance) in 20 cases (25.3%).

**Table 1:** Indications for surgical treatment.

	**Number of cases**
UHN worsening + UTI	30 (38%)
UHN worsening with renal parenchyma thinning	29 (36.7%)
UHN worsening + impairment of DRF	14 (17.7%)
UHN worsening + UTI + impairment of DRF	6 (7.6%)
	79 POM

Clinical data, ultrasonography images, scintigraphy scans, and outcome were preoperatively and post-operatively analyzed. The coexistence of other urological anomalies like paraureteral diverticulum were analyzed in order to determine if they were related to the outcome as it is suggested in the literature. Mixed obstructive-refluxing megaureters were excluded. Intraoperative and perioperative complications were assessed according Clavien-Dindo classification. Statistical analysis was performed with IBM SPSS statistics 20.0.

## Technique

Under general anesthesia and with appropriate antibiotic prophylaxis (usually amoxicillin-clavulanic acid 30 mg/k), a cystoscopy with a 9.5 FG *Storz* cystoscope with 5F working channel is done. For some early cases of the series a retrograde pyelography was performed before dilation, using contrast through a 3 FG ureteral catheter.

**Figure 1:**
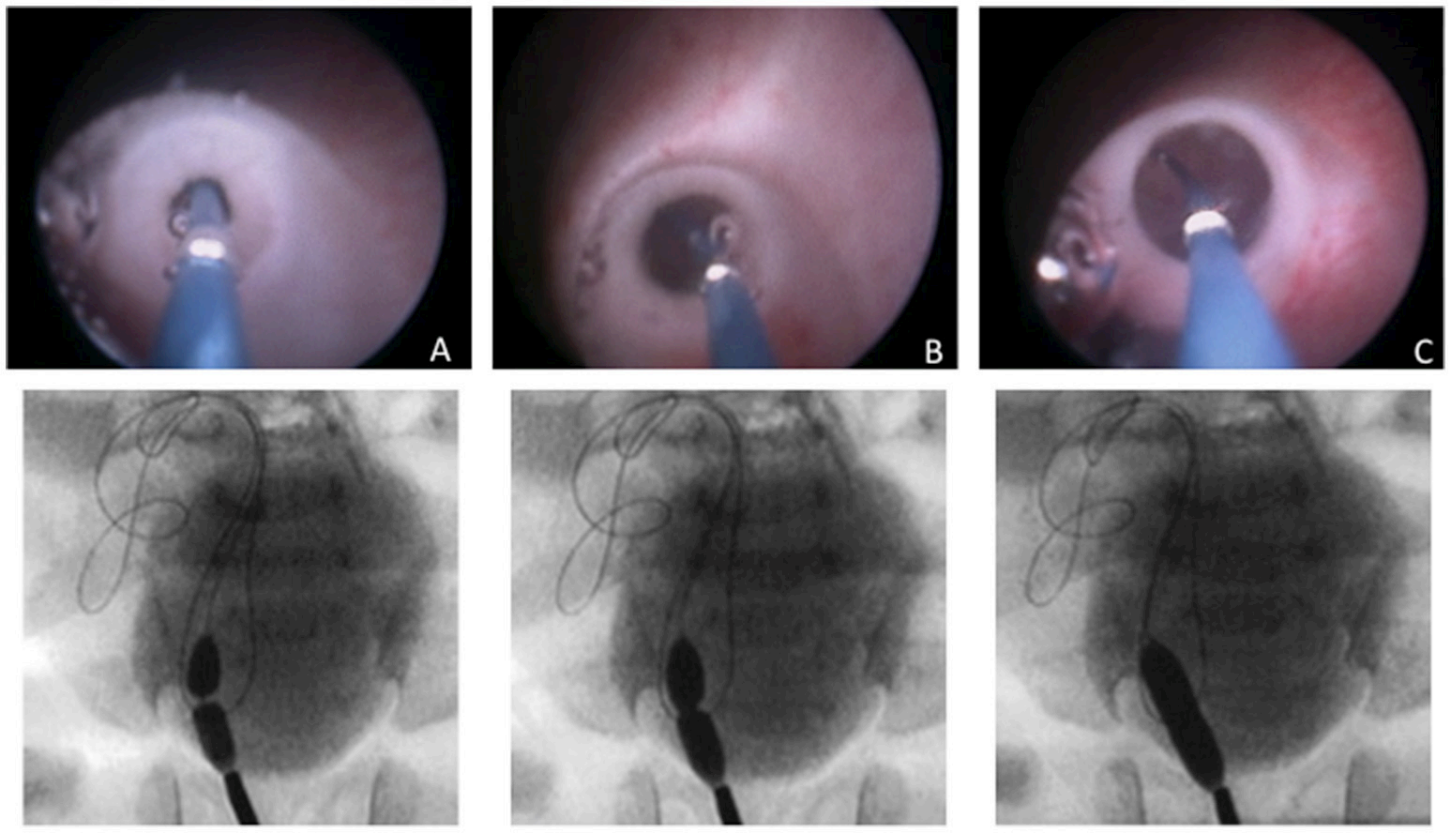
Balloon inserted through the right VUJ, endoscopic view and radiographic control during EBD procedure. **(A)** Initial balloon inflation with presence of stenotic ring; **(B)** Progressive dilation; **(C)** Complete expansion of the balloon and disappearance of the stenosis.

A hydrophilic guidewire (0.014″ *Choice PT*™, *J-tip, Boston Scientific*) or (0.018″ *Radiofocus*® *Terumo)* is introduced through the VUJ, followed by the dilating balloon. The balloons used were semi-compliant dilation catheters with a size of 3.1 F and a nominal diameter from 5 mm to 7 mm and 2 cm length *(RX Muso*™*, Terumo)*. When the balloon is located at the VUJ it is filled with radiologic contrast to its nominal pressure (14 atm) with a pressure inflation device, under direct and fluoroscopic control until the release of the stenosis. Successful dilation is considered when the stenotic ring completely disappears, and the balloon is removed immediately after. Figure 1 illustrates the typical endoscopic and radiology sequence of dilation images.

**Figure 2:**
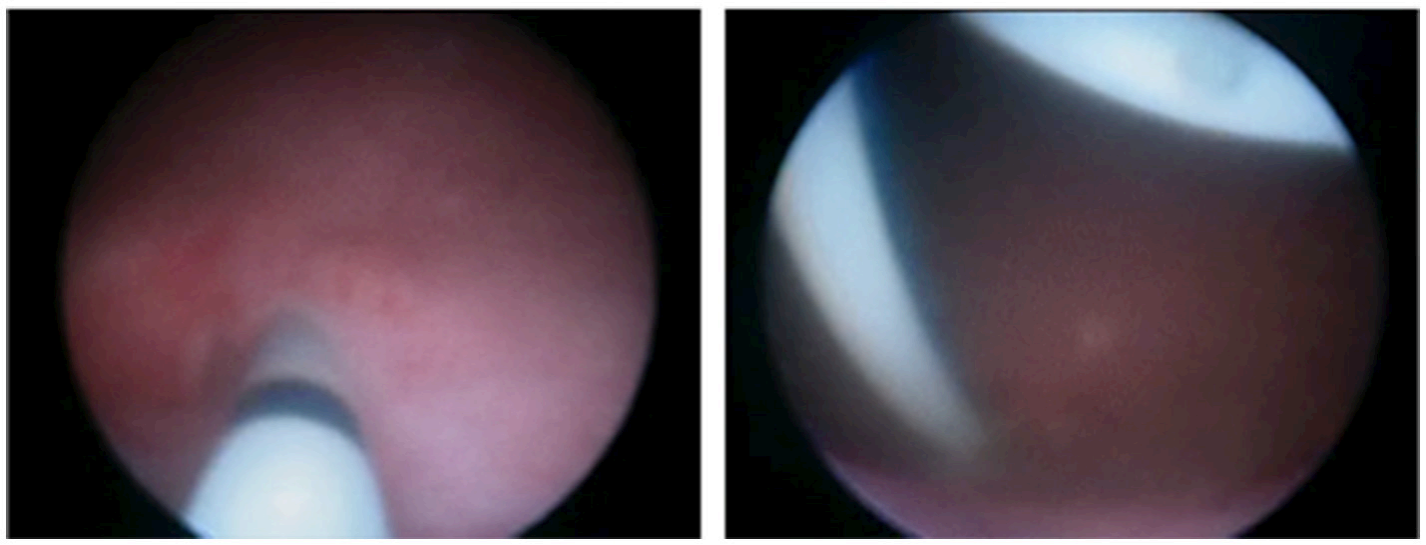
Double J stent placement after EBD of the VUJ.

When dilation is done, the cystoscope is introduced through the distal ureter to assess the VUJ and a double J stent is left *in situ*. In infants under 1 year old we used to place 3Fr, 8–12 cm long *Sof-Flex Multi-Length Ureteral Stents;* in children between 1and 3 years old 3Fr-14 cm long stents and over 3 years 4.8Fr-16 cm*; Cook Medical Europe*™)(Figure 2). After the procedure, a bladder catheter is placed during 24 h to prevent complications.

Double-J stents are removed 4–6 weeks later by cystoscopy. At this time the VUJ is calibrated by distal ureteroscopy. When the cystoscope could be introduced through the VUJ it was considered a satisfactory result. If the VUJ could not be entered with the cystoscope at the time of the Double-J stent removal a new balloon catheter was introduced and inflated at low pressure (2–4 atm) to assess the VUJ diameter. This procedure is considered a calibration of the VUJ and not a new dilation procedure; therefore no further double J stent intervention is needed.

After several years performing this technique, we have done some modifications in order to achieve an easier and shorter procedure, avoiding unnecessary radiation in the majority of cases. Performing a retrograde uretero-pyelography through a narrow meatus may be challenging and it results in mucosal inflammation, oedema or bleeding that may complicate the subsequent procedure. For this reason since year 2011, we have been doing the balloon dilation without fluoroscopic control, only under cystoscopic vision. Retrograde pyelography and fluoroscopic guidance is then reserved for those cases in which the upper urinary tract anatomy needs to be checked, dilation is being difficult or when placing the double J stent is troublesome. With the same purpose, currently the guide-wire and the double J catheter are not meant to reach the renal pelvis, but they are left in the dilated ureter instead. Overcoming the ureteral loops may be technically demanding, time consuming and it implies unnecessary radiation exposure for the baby.

## Follow-up

All children underwent a standard follow-up protocol after endoscopic treatment. This included a clinical review and US at 3, 6, 12, and 18 months after double-J stent removal, and then annually. MAG-3–furosemide renogram scan was performed at 6 and 18 months. Antibiotic prophylaxis was usually stopped in the 6th month after post-operative renogram reveals adequate drainage.

**Figure 3:**
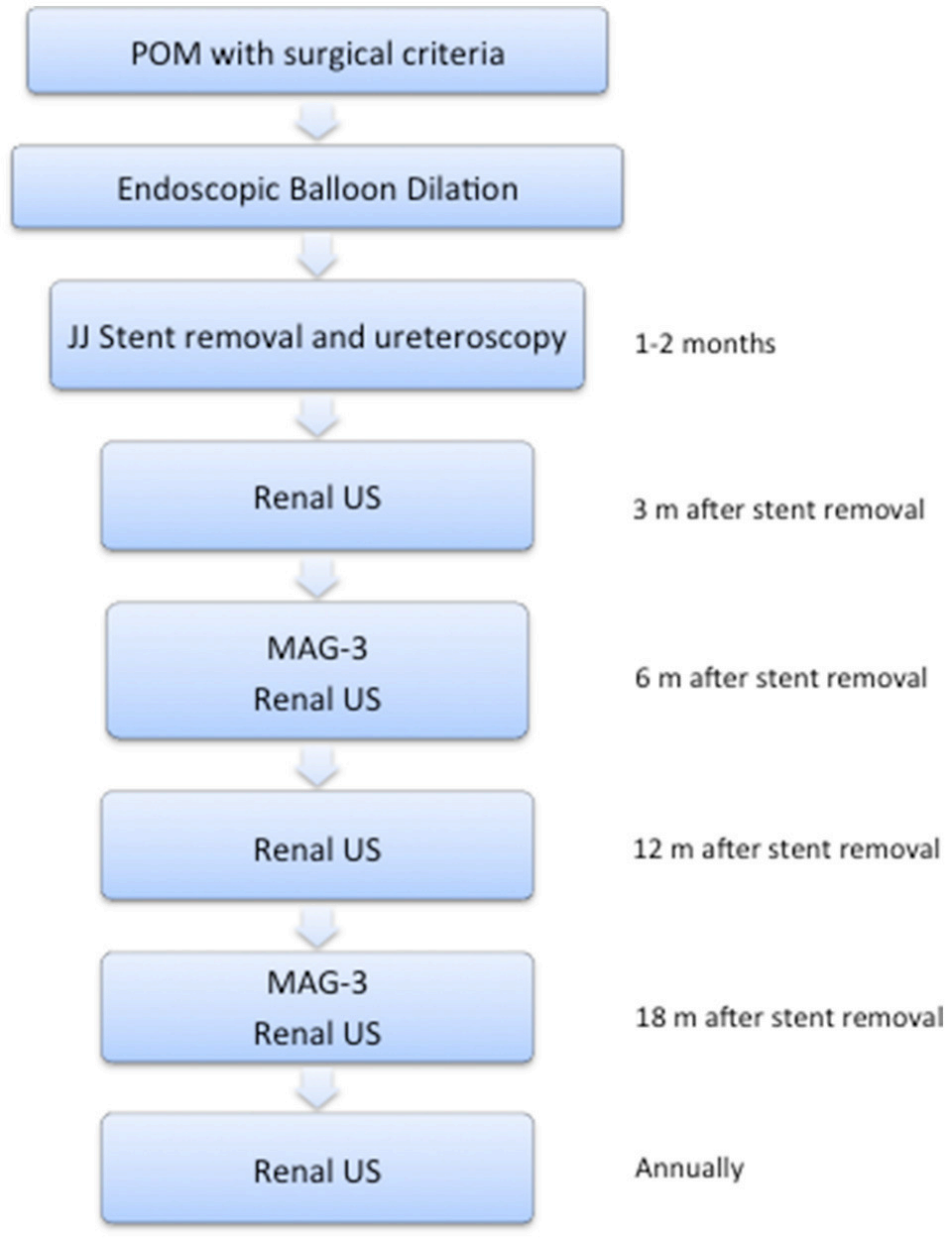
Follow-up protocol. Clinical review and US were done at 3, 6, 12, and 18 months after double-J stent removal, and then annually. MAG-3–furosemide renogram scan was performed at 6 and 18 months.

Voiding cystourethrography (VCUG) was indicated only if patients presented UTI, or non-improvement in ureteral and renal pelvis diameter in post-operative ultrasounds without obstruction at the renogram (Figure 3).

## Results

Median age at surgery was 4 months (0.5–44), with median operating time of 20 min (10–60) and median hospital stay of 1 day (1–7). All patients had hospital admission of 24 h except three patients in whom the endoscopic approach was done at time of urinary sepsis with uretero-pyonephrosis, requiring further medical assistance after the procedure.

There were no intraoperative complications in 75 cases 94.9%. In the other 4 cases (5.1%) EBD could not be performed because of failure of the guidewire to pass through the VUJ in 2 children (ages 6 and 16 months) who went on to successful open ureteral reimplantation. Unsuccessful dilation with false path occurred in the other 2 (ages 15 days and 4 months) requiring temporary nephrostomy and then open ureteral reimplant. The patient of 15 days of life was a single kidney baby treated in obstructive renal failure.

Early perioperative complications occurred in 6 cases (7.8%). Febrile UTI after endoscopic procedure or after double-J stent was removed was reported in 5 patients (Clavien-Dindo 1). One patient presented ureteral double J stent migration and developed early severe re-stenosis with pyonephrosis, requiring nephrostomy (Clavien-Dindo 3) and subsequent open ureteral reimplantation weeks later. Calibration of the VUJ at time of stent removal could not be done with distal ureteroscopy in 11 cases, being performed with balloon catheter inflated at low pressure.

Looking at US findings in patients who had successful initial endoscopic treatment (74/79), significant improvement was observed in hydro-ureteronephrosis in all cases except those who developed re-stensosis or high grade secondary VUR during follow-up. Hydronephrosis grade and ureter diameter showed significant differences before EBD, in the first post-operative US and in long-term (*p* < 0.001 *T*-test). Renal calyces diameters were only measured in 28 patients that also showed significant decreasing in dilation grade after treatment (*p* < 0.05 Wilcoxon test). Renal parenchyma thinning was evident in 49 cases, presenting a progressive improvement after EBD (*p* < 0.001 *T*-test) (Table 2).

**Table 2:** Renal US findings after successful EBD.

	**Preoperatory**	**Early p.o. US**	**Long-term p.o. US**	***p*-value**
Mean pelvis diameter (mm) (*n* = 74)	19.2 ± 4.9	10.3 ± 5.5	5.2 ± 3.5	< 0.001 (*T*-test)
Mean ureteral diameter (mm) (*n* = 74)	14.9 ± 2.9	9.2 ± 4.2	6.6 ± 6.5	< 0.001 (*T*-test)
Median calyces diameter (mm) (*n* = 24)	12.5 (8–21)	9.5 (0–14)	5 (0–10)	< 0.05 (Wilcoxon test)
Mean parenchyma thickness (mm) (*n* = 49)	4.1 ± 1.6	5.5 ± 2.2	8.3 ± 2.4	< 0.001(*T*-test)

*Early postoperative US was performed 3 months after JJ stent removal. Long-term postoperative US was the last on the follow up (1.5–13.5 years)*.

Statistical analysis revealed significant differences in renal drainage on the MAG-3 diuretic renogram before and after endoscopic treatment (T1/2> 50 min vs. 9.8 ± 4.5 min, *p* < 0.001 *T*-test) and in the renal function (mean DRF 44.4% ± 6.3 vs. 46.2 ± 5.9 *p* < 0.05) with no subsequent function deterioration in any case.

Post-operative secondary VUR was found during long-term surveillance in 17 cases (21.5%), being diagnosed in 12 cases after UTI and in 5 after a routine cystography indicated for contralateral VUR previously diagnosed in the initial study of the POM. Only 1/11 patient who needed VUJ calibration with balloon at time of stent removal presented post-operative reflux. Subureteral endoscopic injection of *Deflux*™ *(dextranomer copolymer in hialuronic acid*) was successful in 13 patients (76.4%), and failed in 4 (23.6%) who received an open ureteral reimplantation.

Long-term re-stenosis occurred in 9 cases (12.2%). A new EBD procedure was successfully done in 8 cases (88.9%) at a median post-operative period of 9.5 months (5–63). Only 1 patient developed recurrent re-stenosis and finally required open ureteral reimplantation.

Endoscopic approach of POM including endoscopic balloon dilation of the VUJ and endoscopic management of 2° VUR had a long-term success rate of 87.3% (69/79) with a median follow-up of 5.6 years (1.5–13.5) (Table 3):

- Single endoscopic balloon dilation (*n* = 48) 60.8%
- Successful endoscopic management of 2° VUR (*n* = 13) 16.4%
- Successful endoscopic dilation in re-stenosis (*n* = 8) 10.1%

Endoscopic management of POM failed in 10 cases (12.7%) that required open ureteral reimplantation (Table 3):

- Five were early failures, due to unsuccessful EBD procedure in four and early re-stenosis with JJ migration and pyonephrosis in one.
- Five late failures consisting on persistent 2° VUR in 4 and one re-stenosis recurrence. None of these five children needed ureteral tapering at the open procedure.

**Table 3:**
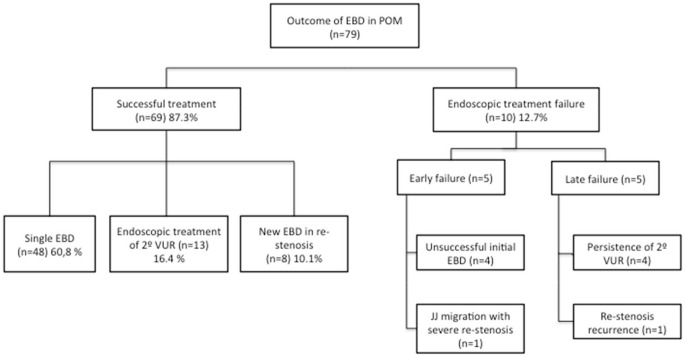
Outcome of endoscopic management in POM.

If we obviate secondary VUR and focus on the final result of EBD as treatment for ureteral obstruction, the long-term results for normalization of urinary drainage and preserving initial renal function was 92.4% (73 of 79).

A total of 43/79 POM were treated under the original technique with fluoroscopic control until year 2011. Since then, 36/79 cases were initially treated without radioscopic guidance, and double J catheters were left on the dilated ureter. Statistical analysis (Spearman's correlation test) did not revealed significant differences between both groups in initial technical failure (*r*:−0.021, *p* > 0.05), early post-operative complications (*r*:−0.028, *p* > 0.05), secondary VUR (*r*:0.052, *p* > 0.05), re-stenosis (*r*:0.011, *p* > 0.05), long-term ureteral reimplant (*r*:0.032, *p* > 0.05), and final outcome (*r*: −0.043, *p* > 0.05) (Table 4).

**Table 4:** Comparison between fluoroscopic vs. no fluoroscopic guidance during EBD.

	**Initial failure**	**Early p.o. complication**	**2^°^VUR**	**Long-term re-stenosis**	**Long-term ureteral reimplant**	**Final outcome (failure/success)**
Fluoroscopic (*n* = 43)	2	3^*^	10	5	3	6/37
No fluoroscopic (*n* = 36)	2	3	7	4	2	4/32
	4	6	17	9	5	10/69

*Spearman's correlation test p > 0.05*.

* *Early postoperative complications were 5 UTI (2 in both groups) and 1 severe re-stenosis with JJ stent migration and pyonephrosis who required open ureteral reimplantation (fluoroscopic group)*.

Attending to the 4 cases of initial technical failure, in 3 of them a severe stenosis of the distal ureter longer than 2 cm in length was evident during the posterior open ureteral reimplantation. The other patient was a 15-day-old baby with a single kidney, treated in a situation of oligoanuria and acute obstructive renal failure.

In 12 cases an ipsilateral para-ureteral diverticulum coexisted with the POM. Ten of them were successfully treated by EBD showing good outcomes in log-term; nevertheless, ureteral reimplantation was required in 2 cases (one persistent VUR and the case of recurrent re-stenosis).

## Discussion

The pathophysiology of the primary obstructive megaureter remains unclear (12). Serial ultrasound studies in prenatal and postnatal periods have helped to better understanding the natural course of POM. These suggest that POM resolves spontaneously in more than 70% of cases without impairment in renal function. However, there is a small group of patients who present a progressive hydro-ureteronephrosis worsening with infectious complications and/or deterioration in renal function. These patients benefit from surgical treatment, which is usually indicated in the first months of life (1, 13–15).

Ureteral reimplantation with or without ureteral tapering is considered the gold-standard procedure for these patients, with a well-documented success rate between 90 and 95%. However, reimplantation of a grossly dilated ureter in a small infantile bladder could be challenging and leads to potential complications such us secondary obstruction, vesicoureteral reflux (15–17) and bladder dysfunction (18).

For this reason temporary urinary diversions could be indicated during first months of life, but are not exempt of complications. External ureterostomies may present problems such as infections, skin irritations, and stenosis (19–21). In addition parental tolerance is usually low, demanding early closure. Percutaneous nephrostomies could be done with external tubes but have limited durability in small infants. Internal urinary diversions have become popular as proposed by Lee and Kaefer (22, 23) who perform a refluxing megaureter reimplantation through a small laparotomy during the first months of life. However, it remains a non-definitive open surgery and creates a high grade secondary VUR.

The important development of minimally-invasive techniques achieved in pediatric age in the last years, have led to non-aggressive procedures for the surgical treatment of POM such as the laparoscopic, robotic, or endourological approach (24–28). Nevertheless, we cannot obviate that the main objective of any technique even minimally-invasive must be to obtain similar success results to the gold—standard, or at least good results with less morbidity or complications.

Several authors have postulated the placement of double J ureteral stent as a temporary internal derivation in the initial management of POM, with good outcomes in a group of patients that did not need any more procedure, but controversial results and remarkable comorbidity in some cases. Castagnetti et al. (24) reported adequate ureteral drainage after double J-stent placement avoiding posterior ureteral remodeling at reimplantation. However, in 50% of the cases double J was placed by open surgery, and more than half finally required ureteral reimplantation. Carrol et al. (25) reviewed 31 patients with POM treated by endoscopic insertion of double-J ureteral catheter. Almost all patients improved from hydro-ureteronephrosis and in 15 of them the obstruction was resolved without requiring further surgical intervention. Nevertheless, half of the cases developed renal dysfunction and 35% finally required ureteral reimplantation. The catheters remained for long periods of time (6 months), resulting in secondary complications such as UTI, incrustation of the stent in the ureteral mucosa, stent migration, and ureteral perforation. Farrugia et al. (26) published the result in a group of 16 patients showing improvement in 56% of cases, but presenting an associated comorbidity of 30% (UTIs, lithiasis, migration of JJ-stent), requiring ureteral reimplantation in 6 cases and nephrectomy due to loss of renal function in 2.

Endoscopic balloon dilation was first described by Angulo et al. (3) in 1998 as initial treatment for children with complicated POM. Since then several publications with few patients and short follow-up periods proved that EBD using the original technique or variations of the same principle was a feasible, safe and less-invasive procedure for the initial management of POM with surgical criteria even for very young patients. In 2007, Angerri et al. (4) reported their initial experience with 6 patients in whom urinary obstruction disappeared without associated complications in a median follow-up of 31 months. Torino et al. (5) presented 5 cases treated below 1 year of age, with resolution of the obstruction after a mean follow-up of 23.8 months. Christman et al. (6) reported in 2012 their experience after the treatment of 17 children with a follow-up of 3.2 years. These authors added a laser incision in cases of ureteral stenosis greater than 2 cm and placed two double J-stent in the ureter simultaneously, reporting good long-term outcome with disappearance of hydro-ureteronephrosis in 71% of the series, while the remaining with moderate improvement on ultrasound showed no obstruction in dynamic-MRI. García-Aparicio et al. (7) presented a series of 13 patients with a medium-term success rate of 84.6% (11 of 13), requiring ureteral reimplantation in 3 patients (2 persistence of UHN and 1 high-grade VUR).

Recent publications have focused on establishing long term effectiveness of EBD as definitive treatment of POM, confirming good results with minimal associated morbidity. Romero et al. (9) reported in 2014 the experience of our institution in 29 patients treated until 2010, with a median age at treatment of 4 months and a median follow-up of 47 months. It was concluded that the patients who had a favorable evolution with disappearance of the UHN and adequate renal drainage confirmed by renogram, remained asymptomatic and with stable situation during the subsequent follow-up. Five patients had secondary VUR and three of them were satisfactorily treated endoscopically. Finally, the endourological management of the POM including EBD of the VUJ and treatment of 2° VUR, had a success rate of 86%. Bujons et al. (10) have reported excellent results in 19 patients, with a long-term success of 90% after the initial dilatation procedure and a follow-up of 6.9 years. One patient required a second dilatation due to re-stenosis, and another one endoscopic treatment of 2° VUR, both with good outcome. Casal et al. (11) have just communicated good outcomes in a short series of 13 patients, but with an important median follow-up of 10.3 years [4.7–12.2), asserting the value of balloon dilation as a definitive treatment for POM.

Technical variations to the initial procedure have been proposed with encouraging results. The group of Kajbafzadeh (27) reported in 2007 a long series of patients treated by endo-ureterotomy (ureterotomy and detrusorotomy at 6 h) leaving double-J stent for 1 week, without associated comorbidity and with a complete resolution of ureterohydronephrosis in 71% of cases. Capozza et al. (8) published the dilation of the VUJ with cutting-balloon™ in 3 patients with persistence of the stenotic ring during the previous endoscopic high-pressure balloon dilation, obtaining a complete resolution of the stenosis and good mid-term post-operative course.

Despite the advantages described of EBD, the endourological management of POM remains controversial. The aspects to be discussed focus on secondary VUR, the possibility of re-stenosis and the use of radiation in young patients. Additionally, it is difficult to assess its value as a definitive treatment in POM attending to the short experience reported in the literature.

Regarding secondary VUR, García-Aparicio (29) analyzed it in his group of patients, reporting 27% (6 cases of 22 POM treated). Of these, 2 were treated endoscopically and 2 were treated by ureteral reimplantation. The author concluded that the coexistence of ipsilateral paraureteral diverticulum is a risk factor for developing secondary VUR, however the number of cases was very low (2 of 4) to establish a reasonable conclusion. In the series published by Bujons et al. (10) only 1 case of 19 presented secondary VUR, and it was resolved endoscopically.

In our series secondary VUR was found during long-term surveillance in 17 cases (23%). Endoscopic treatment of it was successful in 13 patients (76.4%), and failed in 4 (23.6%) who required ureteral reimplantation. For these patients with 2° VUR, three had an ipsilateral para-meatal diverticulum and only one required reimplantation. In our experience, the presence of para-meatal diverticulum was not a bad prognosis factor for the endoscopic management of POM, since 10 of 12 cases of the series had good outcome.

Long-term re-stenosis occurred in 9 cases of our series (12.2%). A new EBD was done with good long-term outcome in 8 cases (88.9%) till the date. Only 1 patient developed recurrent re-stenosis and finally required ureteral reimplantation. The role of cutting-balloon™ dilation may be a useful option in these cases. We have recently used it with excellent mid-term outcome in three patients treated at other institutions who developed re-stenosis time after initial EBD of the VUJ. Currently, we reserve the cutting-balloon™ dilation for future re-stenosis or in primary cases when the stenosis is not completely released with the balloon catheter at time of initial EBD.

Another important issue to discuss is the associated ionizing radiation during this technique, since the risk of radio-induced side effects is especially relevant in the pediatric age. In 2008, the Dosimetry and Radioprotection Unit of our institution published a study in 20 patients with PUJO treated by endourological procedure under fluoroscopic control, in order to determine the probabilistic effects induced by the ionizing radiation administered (30). The average effective dose per minute reported was 0.36 mSv, lower than previously published results for this kind of surgery, being the average total risk of fatal cancer induction in any location 0.012%.

Despite the radiation administered in the EBD of POM is very low, we actually don't use fluoroscopic guidance except selected cases as previously described.

Attending to our experience and looking at the literature, we can consider EBD of the VUJ a relatively simple technique, reproducible and with a short learning curve compared to other procedures. However, its success lies in the use of adequate endoscopic material. The selection of appropriate hydrophilic guide-wires (0.014″-0.018 ″), balloon catheters with low profile and double-J stents suitable for pediatric age are crucial both for the success of the technique and to avoid complications.

## Conclusion

Endoscopic balloon dilation has shown to be a safe, feasible, and really less-invasive procedure in primary obstructive megaureter with surgical criteria even in small infants.

In our experience, we can consider it an effective treatment with few post-operative complications and good outcomes that maintains at long-term follow-up. The main complication observed was secondary VUR, nevertheless it did not result in significant morbidity for the patients and it could as well be treated endoscopically with a high success rate.

In comparison with the conventional surgery, EBD has the obvious advantages of being a minimal-invasive procedure, with a shorter operating time, immediate recovery and with no patient-age limitations. In our opinion, it may be considered first-line treatment in the management of POM in children, avoiding unnecessary bladder surgery in the vast majority of patients. However, it doesn't invalidate ureteral reimplantation in case of failure.

Despite this is the largest series of endoscopic treatment in POM reported to date, its scientific evidence is low. Prospective comparative studies should be encouraged to provide definitive evidence of this procedure.

## Ethics statement

This study was carried out in accordance with the recommendations of Normas de manejo de pacientes pediátricos, Comité Deontológico Hospital Gregorio Marañón with written informed consent from all subjects. All subjects gave written informed consent in accordance with the Declaration of Helsinki. The protocol was approved by the Comité Deontológico Hospital Gregorio Marañón.

## Author contributions

Endoscopic treatment of primary obstructive megaureter was first described by JA 20 years ago. Since then, he has developed the technique as we know it today, divulging and teaching it to the rest of colleagues. All authors of this article and old colleagues of our institution have participated on the diagnosis, treatment, and follow-up of the patients. All authors substantially participated in the conception, design, and execution of the study.

### Conflict of interest statement

The authors declare that the research was conducted in the absence of any commercial or financial relationships that could be construed as a potential conflict of interest.
